# Recent Advances in Microwave Imaging for Breast Cancer Detection

**DOI:** 10.1155/2016/5054912

**Published:** 2016-12-21

**Authors:** Sollip Kwon, Seungjun Lee

**Affiliations:** Department of Electronics Engineering, Ewha Womans University, Seoul, Republic of Korea

## Abstract

Breast cancer is a disease that occurs most often in female cancer patients. Early detection can significantly reduce the mortality rate. Microwave breast imaging, which is noninvasive and harmless to human, offers a promising alternative method to mammography. This paper presents a review of recent advances in microwave imaging for breast cancer detection. We conclude by introducing new research on a microwave imaging system with time-domain measurement that achieves short measurement time and low system cost. In the time-domain measurement system, scan time would take less than 1 sec, and it does not require very expensive equipment such as VNA.

## 1. Introduction

Breast cancer is the most commonly diagnosed cancer in women and has the highest incidence of cancer in women. In 2012, it reached 25.2% of women worldwide. The number of patients who were diagnosed with breast cancer was about 1.7 million worldwide. According to the American Cancer Society, about 246,660 women and 2,600 men will be diagnosed with invasive breast cancer in 2016 in the United States [1]. However, if breast cancer is detected early enough, the five-year survival rate is over 90% [1, 2]. Thus, regular checkups and early detection of breast cancer are crucial.

**Figure 1:**
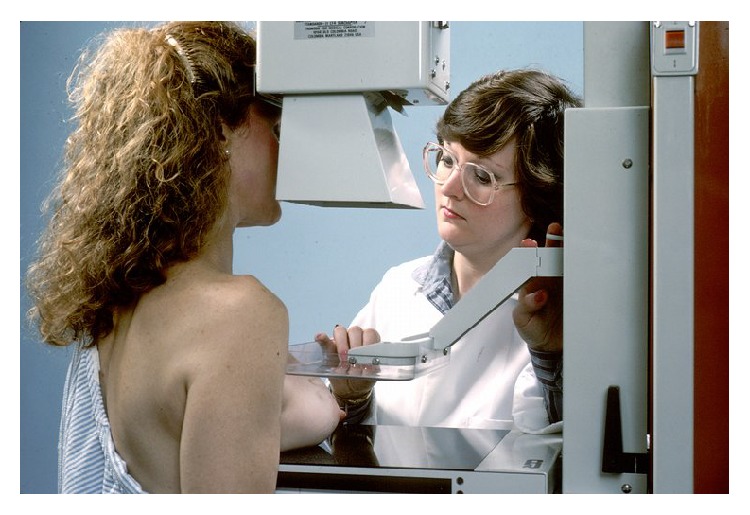
Mammogram examination [7], National Cancer Institute, Public Domain https://commons.wikimedia.org/wiki/File:Mammogram.jpg.

**Figure 2:**
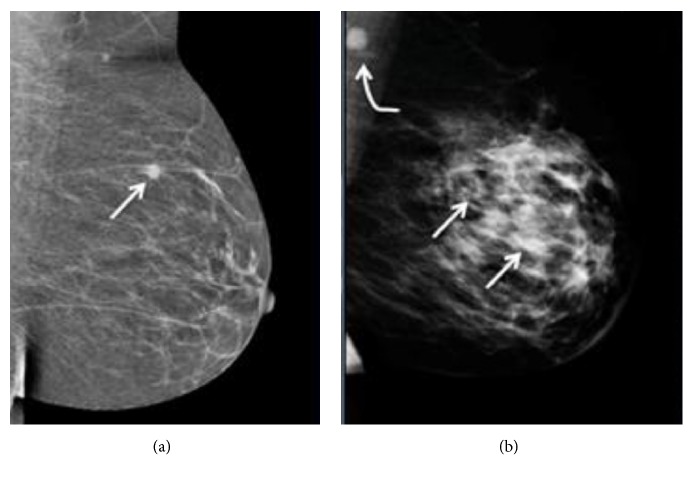
Mammogram images of a fatty breast and a heterogeneously dense breast [8]: (a) fatty breast with a small cancer (arrow), (b) heterogeneously dense breast with a 4 cm cancer (arrows) that is hidden by the dense breast tissue and notable metastatic node left of axilla (curved arrow). © 2015-2017, DenseBreast-info, Inc. and Dr. Regina Hooley.

Current techniques for breast imaging are X-ray mammography, ultrasound, MRI, and positron emission tomography (PET). X-ray mammography uses low-energy X-rays to create images of the breast. It is commonly used for early breast cancer diagnosis. However, it has a number of shortcomings. Several published reports discuss that it risks exposure to radiation [3–6]. The number of women in their 40s experiencing harm from starting regular screening mammography is larger than for older women. The results of mammography revealed 1,212 of 10,000 women screened turn out to be false positives in their 40s. Harmful effects of mammography include overtreatment, unnecessary, and sometimes invasive follow-up testing and psychological harm associated with false positive test results. Further, X-ray mammography needs compression of the breast to lie as flat as possible during the examination (Figure 1), which causes patient pain. It is difficult to distinguish tumors in dense breast mammogram images [1, 6], because both dense tissue and cancer appear white in the mammogram image, unlike fatty tissue that appears black. In Figure 2, a small tumor is conspicuous in a fatty breast; on the other hand, dense breast tissue in a heterogeneously dense breast obscures a 4 cm tumor. Other breast screening methods are adjuncts to mammography.

Ultrasound uses sound waves to see internal body structure. Its frequencies are higher than 20 kHz. A transducer placed on the skin sends ultrasound pulses into the body and detects the echoes from inside the body, which are used to make ultrasound images. This is a painless method that is safe from radiation exposure. However, ultrasound has low resolution and does not distinguish between malignant and benign. Additionally, ultrasound is mostly used as a secondary technique, after a mammogram result shows a suspected mass.

MRI uses radio waves and strong magnetic fields to make images of the inside body. This method utilizes the amount of absorption energy of different type of tissues. When used with soft tissue like breast, contrast liquid is injected to provide better images. MRI is typically used for further evaluation of questionable findings. Moreover, MRI is used for screening evaluation before surgical approach, for example, breast-conserving lumpectomy to mastectomy. However, MRI screening costs are high. Thus, this is barely suitable as an early detection screening method.

PET is an imaging technique that observes how an injection mixture of radioactive materials with glucose or glycoprotein is used and synthesized in the human body. The cancer cells consume nutrients, because those grow much faster than normal cells. When cancer cells consume nutrients, positrons are ejected. PET makes an image by detecting these positrons. Unlike X-ray, CT, and MRI, PET can diagnose cancer in the very early stages, because PET detects the movement of molecules in early disease cells. However, it has low resolution.

Such limitations of existing breast diagnostic imaging methods motivate the research to develop alternative imaging methods. Microwave imaging has been researched as an alternative to X-ray mammography, because of its harmlessness to humans [34–37]. Microwave imaging uses the scattering wave or reflected wave that arises from the contrast in dielectric properties between normal and malignant breast tissues.

In this paper, we review microwave imaging methods to detect breast cancer. First, we review studies of breast tissue properties in the microwave region. Microwave breast imaging could be possible due to the difference in dielectric properties between malignant tissue and normal breast tissue. The following section reviews the results of microwave imaging methods so far. In addition, we introduce our studies of a newly proposed CMOS chip-based time-domain microwave imaging system.

## 2. Breast Tissue Properties in the Microwave Region

**Figure 3:**
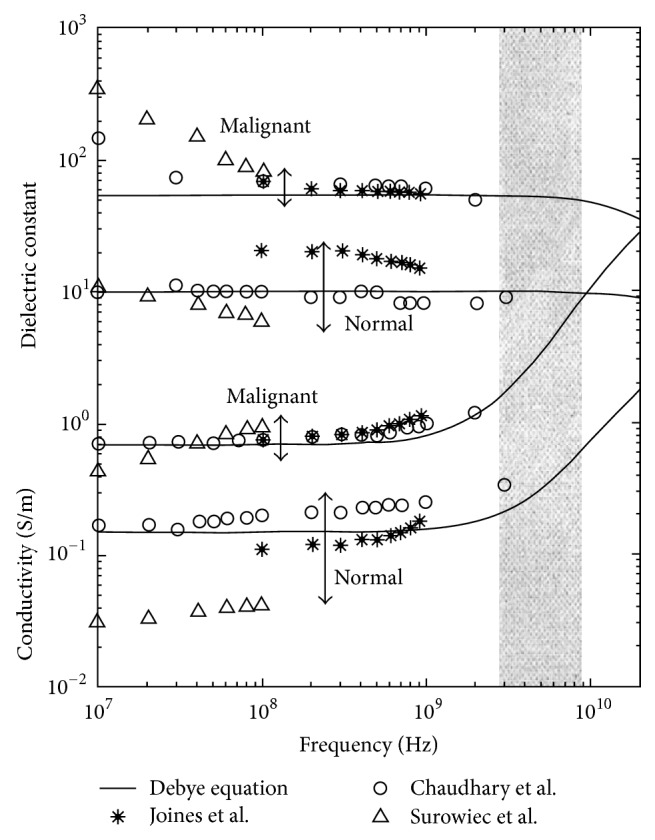
Dielectric properties data for normal and malignant breast tissue at radio and microwave frequencies and single-pole Debye curve fits of measured baseline [9]. Image copyright IEEE, used with permission.

Several groups have studied breast dielectric properties, and research results have already been published [11–13, 38, 39]. The dielectric properties of malignant and normal breast tissues show large differences; and these have been demonstrated by Joines et al. 1980, 1994; Chaudhary et al. 1984; and Surowiec et al. 1988.  Figure 3 shows the dielectric properties of malignant and normal breast tissues from earlier studies. According to Chaudhary et al. 1984, malignant breast tissues at 1~2 GHz are 3~5 times greater than normal breast tissues. The data was obtained from 15 patients. Joines et al. 1994 showed difference between malignant and normal breast tissues at 900 MHz of 200~500%, judging from 12 measurements of each normal and malignant breast tissue. The dielectric properties of malignant breast tissues are approximately ten times as large as normal breast tissues (Figure 3). This is because malignant tissues contain much water and are more active than normal [40, 41].

**Figure 4:**
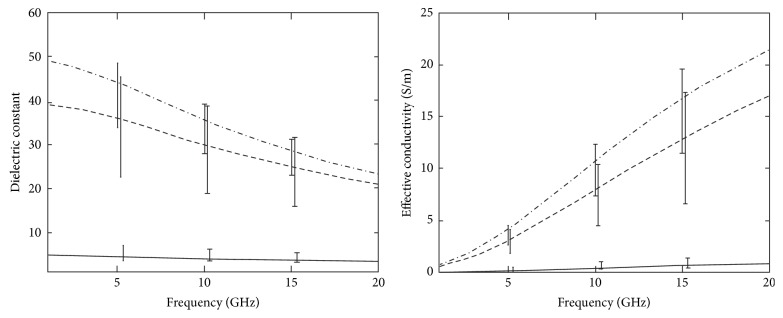
Median dielectric properties of normal breast tissue in three groups categorized by the amount of adipose, from the research team of Wisconsin University and Calgary University. Dash-dot line: group 1 (0–30% adipose), dashed line: group 2 (31–84% adipose), and solid line: group 3 (85–100% adipose) [10]. © Institute of Physics and Engineering in Medicine. Reproduced by permission of IOP Publishing. All rights reserved.

**Figure 5:**
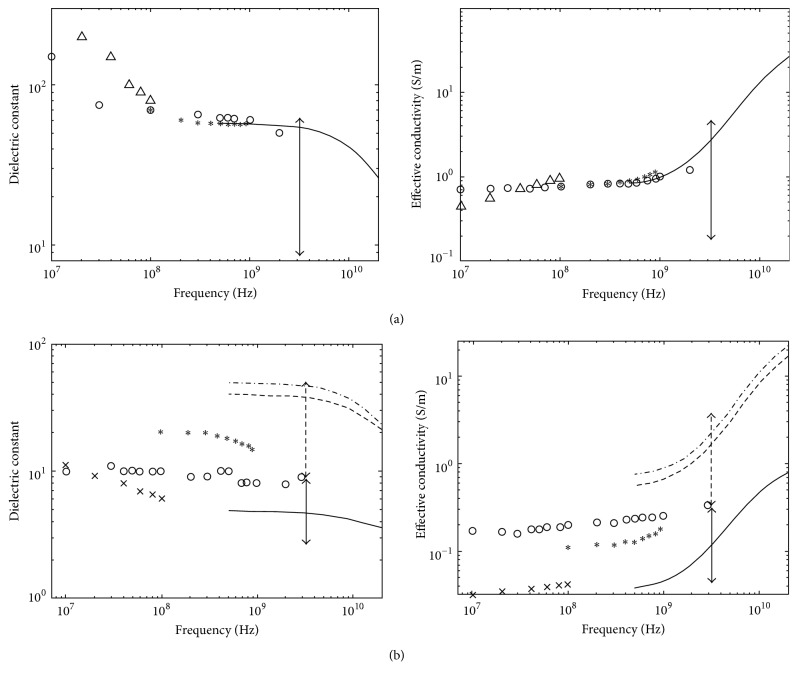
Comparison of malignant tissues and normal breast tissues dielectric properties of previous studies and from the Wisconsin University and Calgary University research team. (a) Malignant tissues properties; (b) normal breast tissues properties. Lines: median dielectric properties of the cancer samples from Wisconsin University and Calgary University research team (dash-dotline: group 1, 0–30% adipose, dashed line: group 2, 31–84% adipose, solid line: group 3, 85–100% adipose). Symbols: malignant breast tissue dielectric properties data published previously (°, Chaudhary et al. [11]; Δ, Surowiec et al. [12]; *∗*, Joines et al. [13]). Vertical arrows: range of data reported by Campbell and Land [14] at 3.2 GHz for malignant tissues [10, 15]. © Institute of Physics and Engineering in Medicine. Reproduced by permission of IOP Publishing. All rights reserved.

However, those earlier studies were small-scale studies. Later, a collaborative research team from the University of Wisconsin and the University of Calgary in 2007 reported a large-scale study of the ultra-wideband microwave dielectric properties of normal breast tissue obtained from reduction surgeries and a large-scale study of the ultra-wideband microwave dielectric properties of normal, benign, and malignant breast tissues [10, 15]. The first study gained tissues from reduction surgeries. The total number of patients was 93, and the total number of measurements was 488. In the latter study, the tissues were collected from cancer surgeries. The total number of patients involved in the study was 196, and the total number of samples was 319. The study measured dielectric constant data in the 0.5~20 GHz frequency range and found that while malignant tissues were ten times larger than adipose-dominated tissues, the difference between malignant and normal glandular or fibroconnective tissues was no more than about 10%. Dielectric properties of normal tissues span a very large range, from 0–30% adipose tissues to 85–100% adipose tissues.  Figure 4 depicts the dielectric properties of 85–100% adipose tissues as solid lines, 0–30% as dash-dot lines and 31–84% adipose tissues as dashed lines. Their dissertation work compared dielectric properties with previous studies.  Figure 5(a) shows the dielectric properties of malignant tissue were similar to the results of previous works, unlike the dielectric properties of normal breast tissues in Figure 5(b).

Whereas initial experiments found the differences of dielectric properties between normal breast tissue and malignant tissue to be close to 10 times, large-scale experiments found that the differences were much smaller. Nevertheless, microwave imaging is still used to distinguish breast cancer [42–44]. Besides, breast cancer classification research in microwave imaging technique has been studied in the National University of Ireland [45–52].

**Figure 6:**
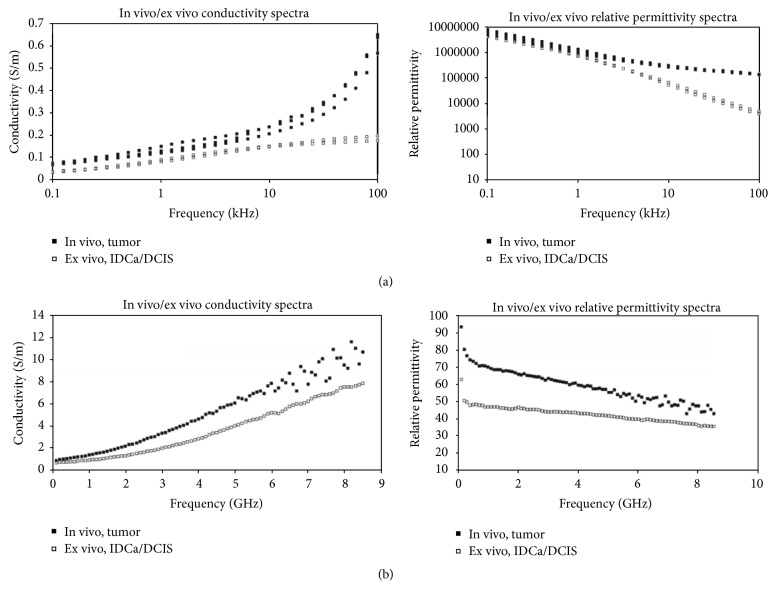
In vivo versus ex vivo dielectric spectra (electrical impedance spectroscopy (EIS) (a) and microwave impedance spectroscopy (MIS) (b)). Multiple spectra recorded for EIS are shown, while the average MIS spectrum is illustrated [16]. © Institute of Physics and Engineering in Medicine. Reproduced by permission of IOP Publishing. All rights reserved.

The research of University of Wisconsin and University of Calgary [10, 15] was conducted at the ex vivo states. Dartmouth College published the correlation of in vivo and ex vivo tissue dielectric-*c* properties which were measured during mastectomy procedure in the operating room (in vivo) and after resection in the same location immediately from six women [16]. The research was carried out to validate the range of properties obtained from the restored images image. Overall, the properties between ex vivo and in vivo are similar, but the properties obtained ex vivo decrease when compared the properties measured in vivo (Figure 6). In addition, the research teams from University Calgary and University Wisconsin have been studying dielectric properties estimation using measurement system [53–57].

## 3. Microwave Breast Imaging

Imaging techniques using electromagnetic waves have been used in specific fields, such as the nondestructive test of structures and detection of hidden objects [58]. Recently, with the technological development of algorithms, computation techniques, and hardware, many studies are being conducted to apply electromagnetic wave imaging technology to human diagnosis [17, 59, 60].

Microwave imaging is a promising new method for early-stage breast cancer detection. This is based on the contrast of electric parameters between the tumor and the normal breast tissue within the microwave spectrum. The method reconstructs the breast image from the received signals that are scattered and reflected within the breast. While the resolution becomes higher as the frequency increases, tissue loss increases. Thus, it is difficult to obtain a clear image, so a limit of upper frequency of the band is needed for acceptable penetration into the tissue. An ultra-wideband signal is appropriate for these conditions. Since the penetration loss of healthy fat tissue is less than 4 dB/cm with microwave signal which is centered at 6 GHz, it is possible to reach low power signal to an antenna on the other side of an object.

The specific absorption rate (SAR) is normally used when setting the safety standards for the maximum allowed exposure to the human body. SAR means the amount of power to be absorbed in the tissue per volume. Standard C95.1-1999 states that, in the case of devices operating at 100 kHz to 6 GHz, it should not exceed a maximum 1.6 W/kg for human tissue of the average 1 g [61]. As a guide, compared to a mobile phone using the same frequency band, microwave breast cancer imaging method should be free from health hazardous exposure to the patient, because of suffering less exposure than from a mobile phone.

From the technical point of view, microwave breast cancer imaging method may detect a small size tumor and also has the advantage of low cost. In addition, microwave imaging can obtain an image quickly compared to other imaging methods [17].

There are two approaches to the microwave imaging method: microwave tomography and radar-based. Both approaches use the scattering of microwave signals. The systems transmit the microwave signals into the breast and then measure scattering signals reflected from the tumor.

### 3.1. Microwave Tomography

Microwave tomography has been studied by several research groups [18–20, 62–85]. Prof. Paulsen and Prof. Meaney from Dartmouth College in the United States are one of the representative research groups. They have studied microwave tomography breast imaging since 1990s [86–89]. They proposed an iterative reconstruction algorithm that obtains dielectric properties in a 2D lossy medium in 1995.

The goal of microwave tomography is to recover the profile using the inverse problem of the dielectric properties of the breast. Microwave tomography uses an inverse scattering method to get a breast diagnostic image. Inverse scattering uses scattering signals including diffraction from objects. It creates a map of permittivity and conductivity through inversion of those signals. However, the inverse problem takes much time, because the calculation process is complicated. Also, a nonlinear inverse scattering problem must be solved, and iterative image reconstruction algorithms are usually required to obtain a solution. In general, these ill-posed inverse scattering approaches suffer from nonuniqueness and require regularization in order to achieve convergence to a meaningful solution [66, 90].

**Figure 7:**
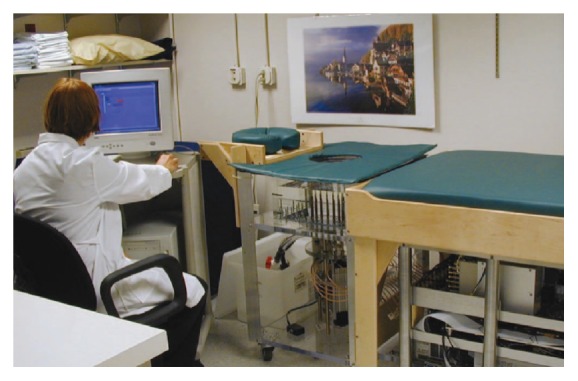
The microwave breast imaging system at Dartmouth College [17]. Image copyright IEEE, used with permission.

**Figure 8:**
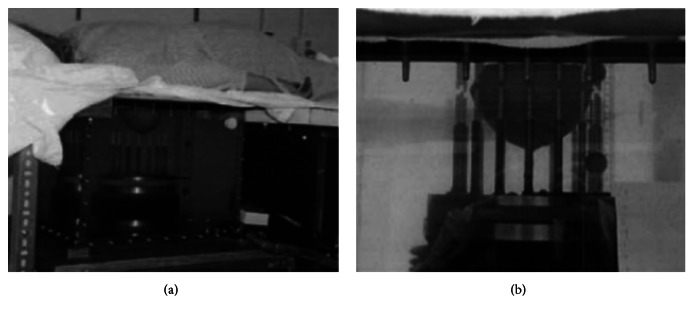
Photographs of a microwave exam with patient in Dartmouth College. (a) Close-up view, and (b) the breast in position within the center of the antenna array [18]. Image copyright IEEE, used with permission.

**Figure 9:**
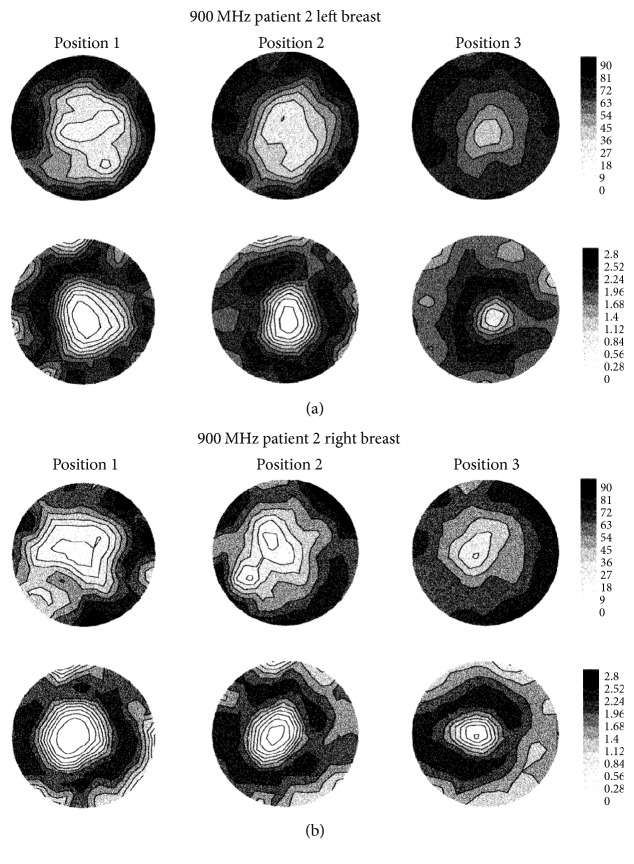
Recovered 900 MHz relative permittivity and conductivity images from patient (a) left breast and (b) right breast [18]. Image copyright IEEE, used with permission.

The Dartmouth group has developed a clinical prototype for active microwave imaging of the breast for the first time [18]. They developed a 32-channel data acquisition system operating at frequency range 500 MHz to 3 GHz [87] to obtain data from a clinical prototype exam. The clinical prototype illuminates the breast with 16 monopole antennas that operate in the 300 MHz to 1 GHz frequency range. The clinical exam was conducted with 5 women, and the total acquisition time was 10–15 minutes per breast. The system measured data at seven different array heights. The data were obtained from seven different array heights for seven different frequencies at each array position.  Figure 7 shows the tomographic microwave breast imaging system at Dartmouth College.  Figure 8 shows the woman being screened using the clinical prototype and Figure 9 shows the resultant images.

**Figure 10:**
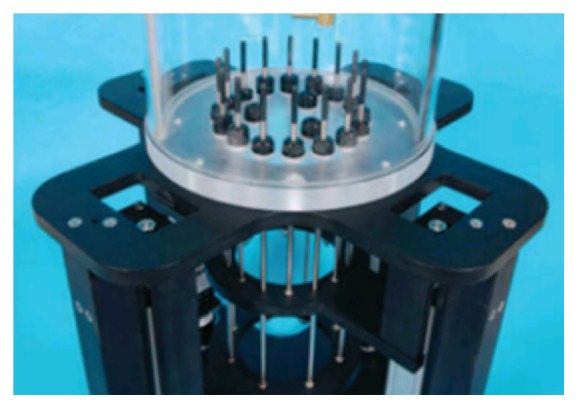
The 3D microwave tomographic breast cancer imaging system of Dartmouth College [19]. Image copyright IEEE, used with permission.

**Figure 11:**
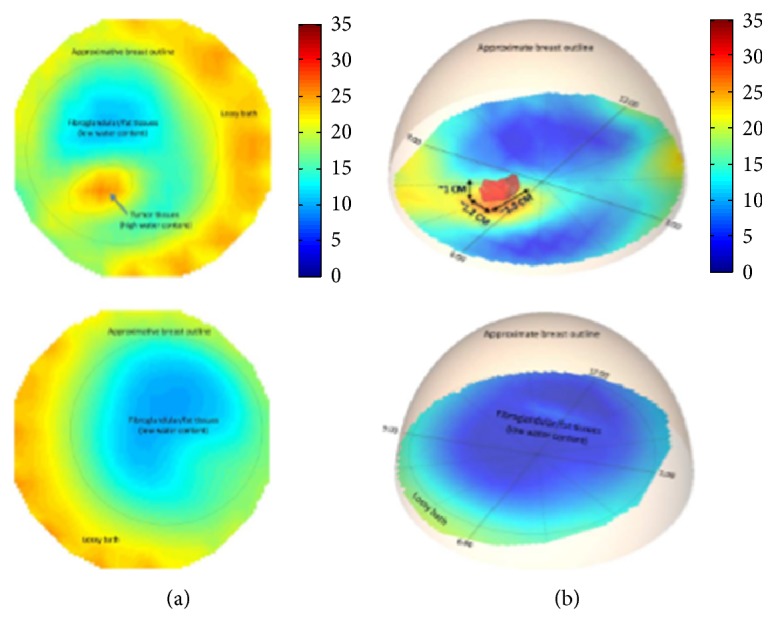
Microwave tomographic permittivity images at 1300 MHz from Dartmouth College; upper: right breast; lower: left breast. (a) 2D images and (b) 3D images [20]. Image copyright IEEE, used with permission.

They have also developed a prototype of breast cancer detection system based on 3D FEM method microwave tomography [19, 20].  Figure 10 shows the prototype with computer-controlled arrays. They presented the first clinical 3D microwave tomographic images of the breast using the prototype [20]. The study achieved improvements of both hardware and software aspects and achieved data acquisition time of less than 2 minutes by development of special hardware. The image processing time was within 20 minutes. This is very fast compared to ten of hours or even days for a single 3D microwave tomographic image [91].  Figure 11 shows the 2D and 3D tomographic permittivity images [20].

They used the 3D microwave breast imaging system for monitoring treatment response during neoadjuvant chemotherapy [20, 92]. Tomographic images were obtained eight times from eight patients undergoing neoadjuvant chemotherapy during the course of treatment [92]. The results of the study indicate that the conductivity in locally advanced breast cancer matched well with overall neoadjuvant therapy treatment response [92].

**Figure 12:**
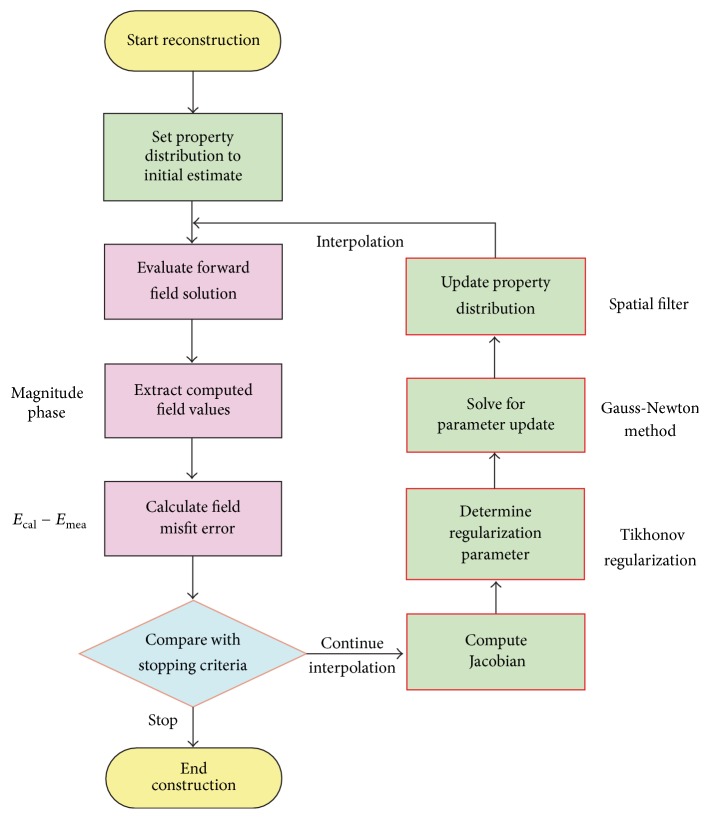
Flow chart of the ETRI reconstruction algorithm [21]. Image copyright ETRI, used with permission.

**Figure 13:**
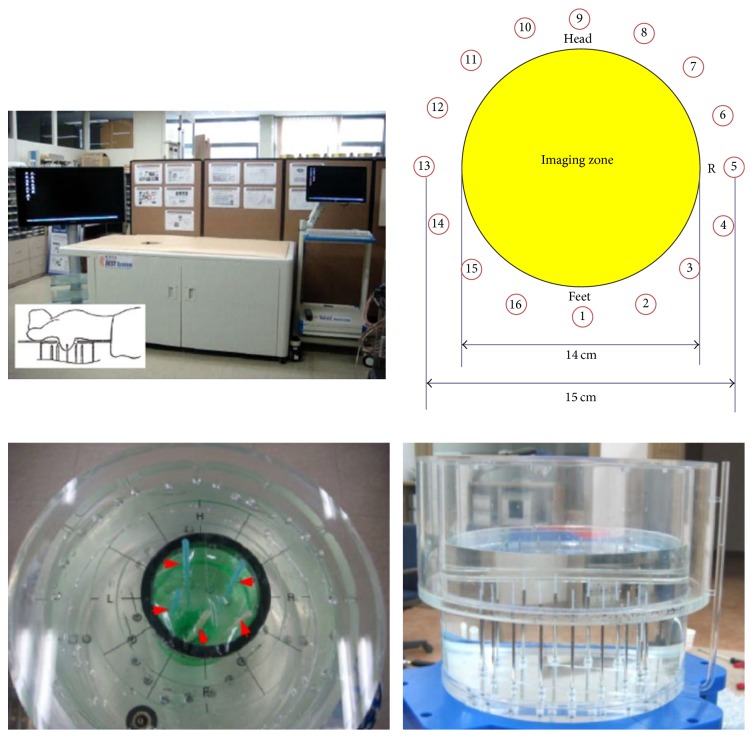
ETRI microwave tomography system [21]. Image copyright ETRI, used with permission.

**Figure 14:**
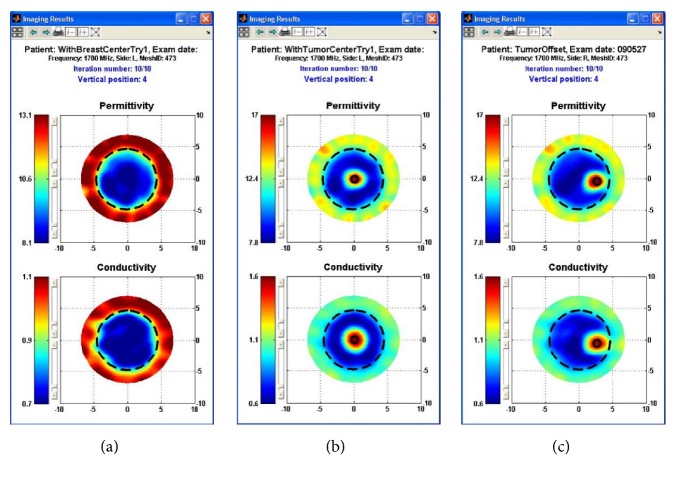
Reconstructed images of cylindrical tumor (10 mm diameter pipe) inside bath liquid at ETRI. (a) Breast without tumor (b) with tumor at center and (c) with tumor 30 mm right of center [21]. Image copyright ETRI, used with permission.

In Korea, Electronics and Telecommunications Research Institute (ETRI) started research of the breast cancer diagnostic technology of microwave tomography in 2007. ETRI MT breast cancer diagnostic techniques have been studied using a microwave signal in the 500 MHz to 3 GHz range [21, 78, 93].  Figure 12 shows a flow chart of the ETRI iterative reconstruction algorithm [21].  Figure 13 shows the microwave tomographic breast imaging system developed by ETRI. Experimental ETRI MT system consists of cylindrical breast phantom and cylindrical tumor phantoms (Figure 13). The five thin-wall plastic pipes displayed here are filled with the liquid of complex permittivity similar to a real tumor.  Figure 14(a) shows reconstructed images of a breast without a tumor, and Figures 14(b) and 14(c) present images of a single cylindrical tumor at the central and shifted positions [21].

**Figure 15:**
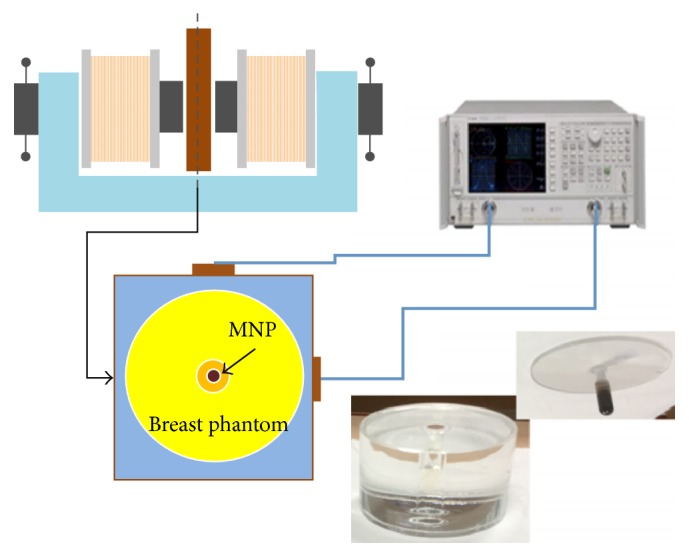
Experimental setup of IREA, National Research Council of Italy for MNP enhanced microwave imaging [22]. Image copyright IEEE, used with permission.

King's College in London has been developing antenna for microwave tomography [84, 94], and they applied an adaptive multithreshold iterative shrinkage algorithm to the linear inversion at each iteration of the Distorted Born Iterative Method (DBIM) [95]. Some groups are researching on reducing high computational demands due to a number of iteration and discretization of the object image for accuracy. Xu et al. in University of Manitoba proposed an iterative process that involves two algorithms: Finite-Difference Time-Domain (FDTD) and Genetic Algorithm (GA). They developed a parallel algorithm for microwave tomography on CPU-based homogeneous multicore distributed memory machines and the Cell BE processor [68, 69, 76]. The research group in National University of Ireland Galway in Ireland presented a novel parallelization strategy to accelerate microwave tomography [85]. IREA, National Research Council of Italy, has developed diagnostic and therapeutic strategies based on the use of electromagnetic fields at microwave frequencies for some years. They have researched magnetic nanoparticles (MNP) enhanced microwave imaging which is able to reduce the rate of false positive and negative [22, 96–98].  Figure 15 shows the experimental setup for the MNP enhanced MWI technique which IREA developed. Microwave ablation monitoring via microwave tomography also has been researched in IREA [99]. In addition, the research has been progressed in therapeutic aspects with microwave ablation at IREA and University of Wisconsin-Madison [100–102].

### 3.2. Radar-Based Microwave Imaging

Microwave radar imaging reconstructs the image using the reflected wave from objects. It uses the reflection that arises due to difference in the electrical dielectric properties of normal and malignant breast tissues, when microwaves transmit the internal breast with tumor. This approach, which was first developed as a military ground-penetrating application, is applied to the human body. It was designed in the late 1990s by Hagness in Wisconsin University, and Benjamin in Bristol University [9, 24, 103–106]. Researchers in National University of Ireland Galway, Tianjin University in China, and Hiroshima University are also working on this subject [107–123]. Recently, Goethe University of Frankfurt and University of Bristol have been developed experimental phantom based on 3D printing technology that was one step close to the development of standard breast phantoms [124].

A 3D image of the received signals through the breast is obtained by focusing algorithms. Before applying the focusing algorithm, preprocessing is performed to obtain a tumor response. Preprocessing may contain the extracting tumor response, compensation tissue losses, or radial spread. Among the preprocessing, extracting tumor response must be conducted, because the received signal includes not only the tumor response, but also unwanted signals such as antenna coupling, directly received signals from the transmit antenna, and reflections from the skin.

**Figure 16:**
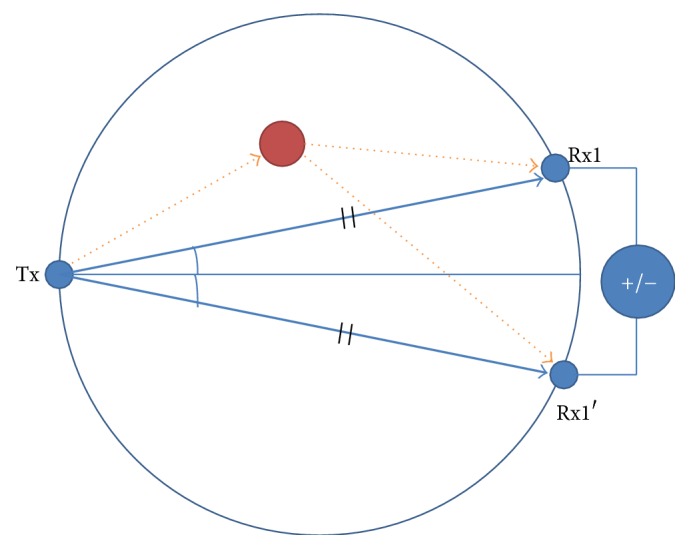
In-place calibration with dual receivers.

Existing techniques to extract tumor response are differential calibration [37] and array rotation calibration [36]. Differential calibration techniques use a reference signal obtained from a scan of a healthy breast [37]. However, it is hard to use when previous knowledge of the cancer-free breast does not exist. In the array rotation calibration method, two sets of measured data are received from a physically rotating antenna array, in which unwanted signals are almost the same [36]. These two methods measure two sets of the signals by multiple measurements. A calibrated signal is obtained by subtracting one of the two sets of the signals from the other. We have proposed a new calibration method called “in-place calibration” to extract a tumor response [125, 126]. The received signal and the reference signal are measured at the same time. In-place calibration eliminates unwanted signals using dual receivers, Rx1 and Rx1′, located an equal distance from the transmitter Tx in Figure 16. Antenna coupling and skin reflection received by the two antennae occur at an identical time, unlike tumor responses that transpire in a different time due to a slight delay. Thus, the calibrated signal of a tumor response can be obtained by subtracting one received signal from the other. In-place calibration reduces the time of measurement. In addition, measurement errors that occur due to environmental changes during calibration, such as a mechanical error during antenna array rotation or an accidental error due to the unexpected movement of the patient, are minimized. Extra hardware for calibration like highly precise motor for rotation is not required. We assumed that the breast shape is hemispherical while a real breast is not exactly rotational symmetric. Various breast shapes other than hemispherical may hinder the in-place calibration method. A possible solution for this is to provide different sizes of hemispherical measurement bowls such that a breast can tightly fit into the bowl during measurement. In addition, there might be mismatches between two antennae. It can be easily compensated once the characteristics of the antennae are obtained [126].

Focusing algorithms that are used for image reconstruction in radar-based microwave imaging include the delay and sum (DAS) [36, 127–129], microwave imaging via space-time (MIST) [108, 128, 130], robust weighted Capon beamforming (RWCB) [131], multistatic adaptive microwave imaging (MAMI) [36, 110], and generalized likelihood ratio test (GLRT) [109, 132].

DAS is a simple and robust method. Also, the computation time is short. The signals are shifted by time *T*_*i*_ calculated from their respective location relative to the antennae, to restore the image. The focal point is assumed to be *p* = (*x*, *y*, *z*). The time delay *T*_*mn*_ is assumed as the time delay of the signal from transmitting at the *m*th antenna to receiving at the *n*th antenna. The time delay *T*_*mn*_ at the focal point p can be expressed as

(1) Tmnp=1vxm−x2+ym−y2+zm−z21/2+xn−x2+yn−y2+zn−z21/2.

If the number of antennae is *N*, the number of signals used by the focusing algorithm is *M* = *N* × (*N* − 1)/2. The time delay applies the received signal rs_*i*_ differently for each transmit antenna and receive antenna. The intensity of a reconstruction image is calculated by moving the focal point from one position to another within the breast. The integration window *τ* is determined by the input pulse width. Normally, *τ* uses 50 percent longer than the input pulse width, due to the antenna effects and dispersion [133]. The intensity *I*_*p*_ of the restored image at the given focal point can be expressed as

(2) Ipx,y,z=∫0τ∑i=1Mwix,y,z·rsit−Tix,y,z2dt,

where *w*_*i*_ is the location-dependent weight calculated during preprocessing, rs_*i*_ is the received signal, and *T*_*i*_ is the time delay.

**Figure 17:**
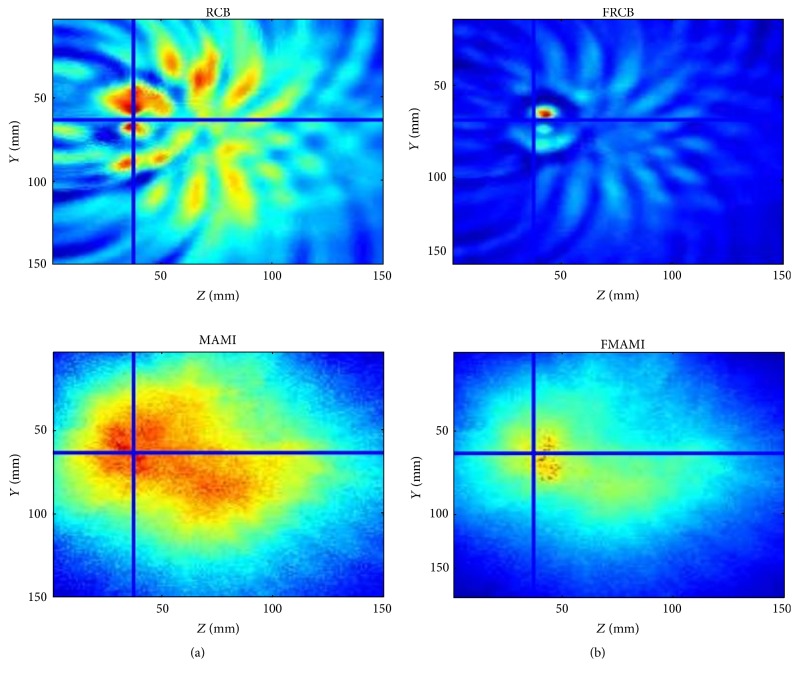
2D slice of reconstructed images from National University of Ireland Galway (a) without prefiltering and (b) with prefiltering [23]. Reproduced courtesy of The Electromagnetics Academy.

Microwave imaging via space-time (MIST) beamforming uses finite impulse response (FIR) filters to compensate for the frequency-dependent time delay, such as dispersion and fractional time delay. The microwave image is formed by summing the filtered signals. Microwave breast cancer imaging uses a generalized likelihood ratio test (GLRT), which is a hypothesis testing problem for each voxel, with the null hypothesis representing the tumor-free case. Reference [109] shows the image formation equations. However, most of those methods have suffered from performance degradation used with dense breast. Thus, in the National University of Ireland Galway they used preprocessing filter to compensate the path-dependent attenuation and phase effects. Consequently, they achieved more clear images (Figure 17) [23]. In addition, they investigated the focal quality metrics to estimate average dielectric properties to enhance tumor detection [51]. University of Bristol also presented a time-domain wideband adaptive beamforming to reduce clutter [134]. The approach uses an adapted equalization filter that adapts a calculated estimation of averaging dielectric properties of the breast.

#### 3.2.1. Frequency Domain Measurement

Radar-based microwave imaging can be done through two approaches: frequency domain analysis and time-domain analysis. There have been several microwave systems reported in the literature, most of which are based on frequency domain analysis [24, 26, 28, 36, 135]. However, frequency domain analysis requires equipment, such as a costly vector network analyzer (VNA). The process takes a lot of time, because it measures *s*-parameters by sweeping frequencies; and measurement time is critical in medical imaging: patient movement during a scan may generate artifacts.

**Figure 18:**
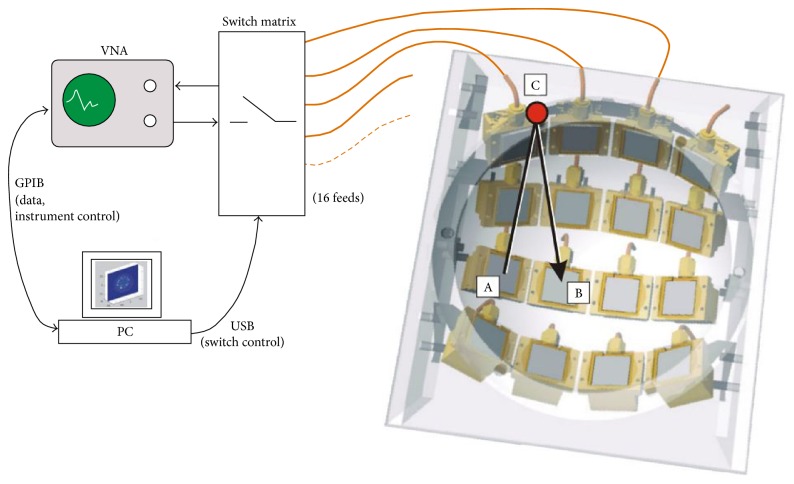
Radar-based microwave imaging system schematic with frequency domain measurement for breast cancer detection at Bristol University [24]. Image copyright IEEE, used with permission.

**Figure 19:**
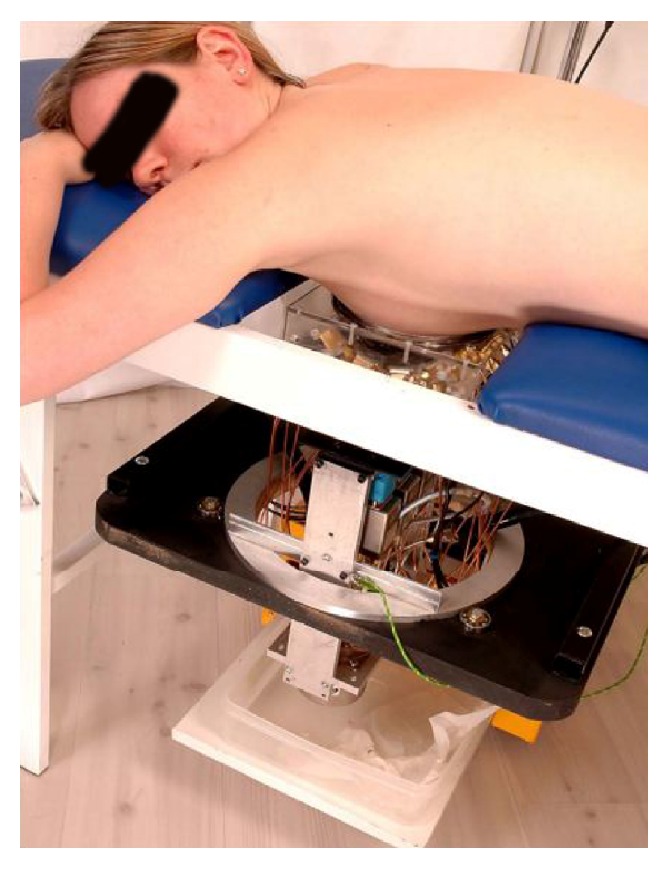
Clinical test with microwave radar-based system at Bristol University [25]. Image copyright IEEE, used with permission.

**Figure 20:**
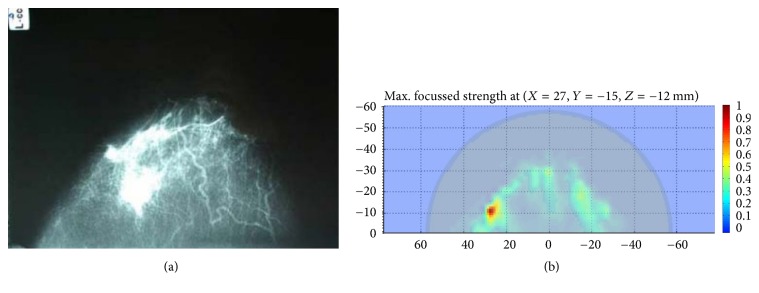
Comparison clinical images of the radar-based microwave imaging and X-ray mammogram result at Bristol University: (a) X-ray mammogram and (b) radar-based UWB microwave system (2D slice through a plane with tumor) [25]. Image copyright IEEE, used with permission.

University of Bristol team performed experimental and clinical study using UWB microwave radar imaging method for the first time [25].  Figure 18 shows a schematic of the multistatic radar-based microwave breast imaging system [24]. Scattered signals are measured in the frequency domain using VNA. A switch matrix connected to the feed of the antennas changes the pair of antennae during measurement of the signals. The signals are measured, in turn, by all possible pairs of antennae that activate the transmit port and receive port. The measured signals with frequency domain are transferred to time-domain signals using an inverse FFT method. The image is reconstructed by a focusing algorithm using the time-domain signals.  Figure 19 shows the microwave radar-based clinical setup for breast cancer detection with a patient. Clinical verification was performed with real breast cancer patients at the Bristol Oncology Center.  Figure 20 shows that the contribution compared images obtained using X-ray mammography and the radar-based microwave system [25].

**Figure 21:**
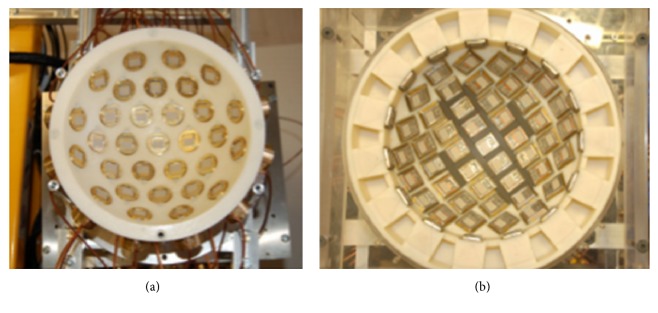
(a) 31-element prototype and (b) 60-element antenna array at Bristol University. Taken from [26]; copyright EurAAP; used with permission.

**Figure 22:**
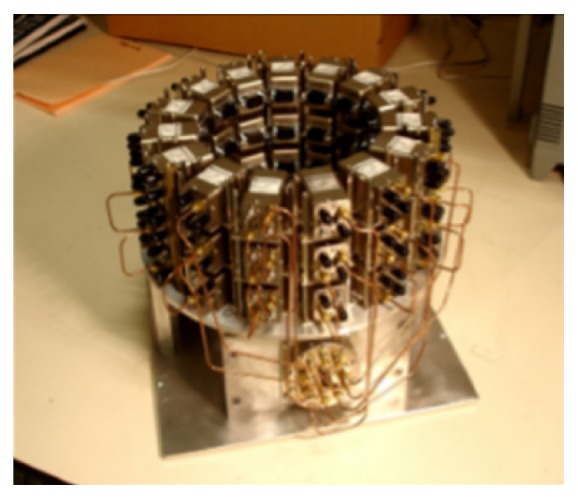
The completed 60-way TX/RX electromechanical switching interface at Bristol University. Taken from [26]; copyright EurAAP; used with permission.

**Figure 23:**
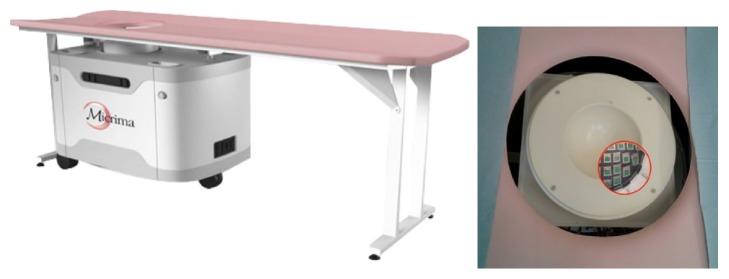
Breast imaging system “MARIA™” of MICRIMA [27]. Image copyright Micrima Ltd.

Figure 21(a) shows the system based around 31 UWB slot antennas called “MARIA” that University of Bristol team developed [42, 135]. It uses a 2 port VNA, and takes approximately 90 seconds to scan. However, 90 seconds is a long time to scan patients, and artifacts can occur due to patient movement. Subsequently, the team developed a 60-element antenna array with 8 port VNA (Figure 21(b)).  Figure 22 shows the electromechanical switching interface for the 60-way TX/RX. The scan time with the 60-element array achieved 10 seconds.  Figure 23 shows the radar-based breast imaging system of MICRIMA which was founded in 2006 to develop and commercialize the technology of microwave radar breast imaging at the University of Bristol in the UK. The MICRIMA's MARIA system has undergone clinical trials at the several breast cancer imaging centers in the UK and got European regulatory approval in 2015 [27].

**Figure 24:**
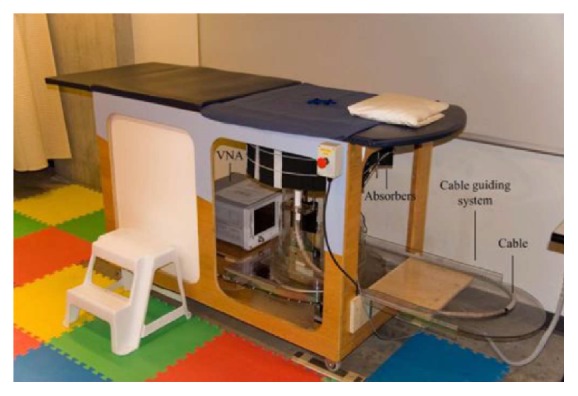
TSAR prototype system in Calgary University [28]. Image from Bourqui et al. 2012, under the Creative Commons Attribution License.

University of Calgary have developed a tissue sensing adaptive radar (TSAR) prototype of radar-based breast imaging system (Figure 24) [28]. The patient lies face down on the table to be scanned and immerses the breast in a tank filled with immersion liquid. Their system was different from the system of Bristol University, using a monostatic method and filtering the reflection from the skin. They scanned a small group of patients using the prototype [136]. The results were positive, since several restored images exhibited responses which were similar to clinical results. They also studied 3D surfaces acquisition of the breast for radar-based microwave breast imaging [137] and investigated the impact of the breast model complexity on microwave imaging signals [138].

#### 3.2.2. Time-Domain Measurement

Several research groups have recently reported time-domain measurement microwave breast imaging systems [29, 31, 37, 139]. Time-domain measurement systems have the advantage of being cost-effective and requiring less scan time.

**Figure 25:**
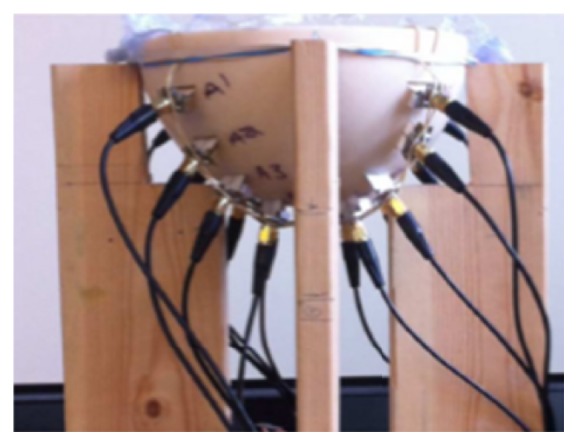
Antenna array at McGill University [29]. Image copyright IEEE, used with permission.

**Figure 26:**
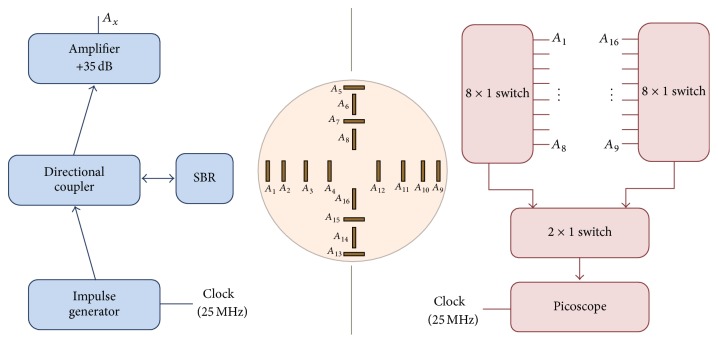
Schematic diagram of the time-domain breast imaging system of McGill University [29]. Image copyright IEEE, used with permission.

**Figure 27:**
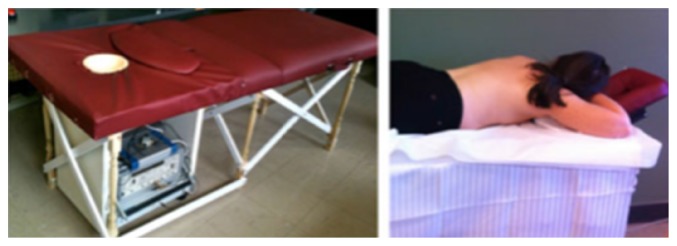
Photos of clinical prototype with test table and scanning patient at McGill University [30]. Image copyright IEEE, used with permission.

McGill University in Canada is a leading research group in time-domain measurement microwave imaging systems for the detection of breast cancer.  Figure 25 shows the experimental system that is a multistatic radar-based microwave system with 16-element antenna array for breast cancer detection that their research team developed [29].  Figure 26 shows that the time system transmits input pulse using a pulse generator and receives the signal from antennae using an oscilloscope in real time [29, 140]. Further, the McGill University research team have performed an early clinical study of time-domain microwave radar for breast health monitoring [30, 141]. They collected a total of 342 breast scans over an eight-month period with 13 healthy volunteers for monthly scans [141]. The purpose of the study was to investigate the impact of unavoidable multiple factors of human in monthly monitoring.  Figure 27 shows a scanning patient on the table with the clinical system at McGill University. In addition, they have developed wearable bra prototype using microwaves for breast health monitoring [142, 143].

However, the system still depends on a high-precision pulse generator and very high-speed oscilloscope and may require complicated switching circuits. Additionally, a time-domain system requires a very fast sampling clock such that even a very small jitter in the sampling clock might blur the resulting images [144].

**Figure 28:**
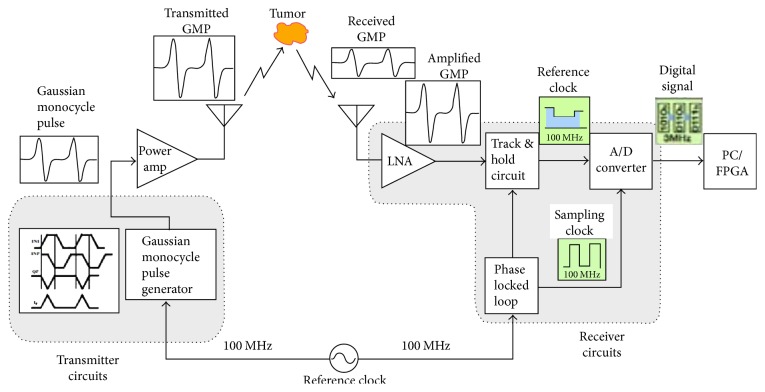
A Hiroshima University concept for a CMOS breast cancer detection system [31]. Image copyright IEEE, used with permission.

**Figure 29:**
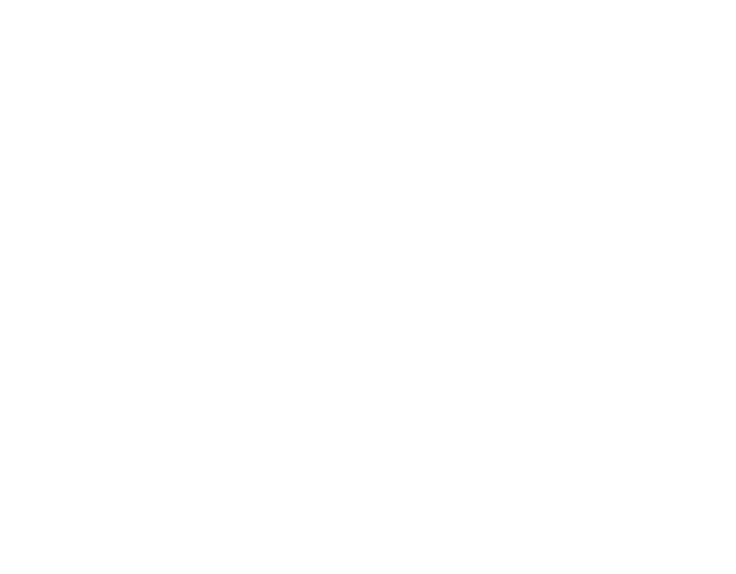
This figure has been blanked, since it was reproduced from [32] without permission from the Institute of Electronics, Information and Communication Engineers (IEICE).

**Figure 30:**
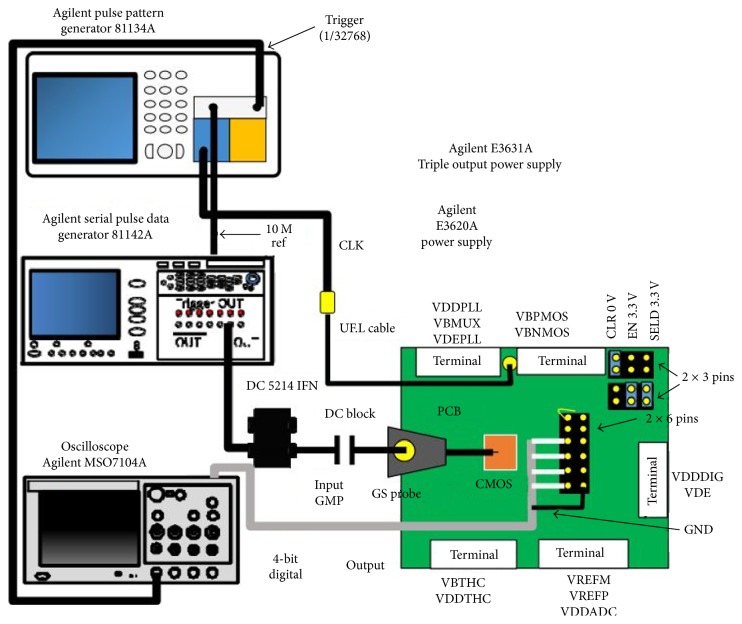
GMP equivalent time sampling measurement setup of Hiroshima University [33]. Image copyright IEEE, used with permission.

Figure 28 shows the system implementation using CMOS circuits as an alternative to high-speed pulse generator and oscilloscope proposed by Hiroshima University in Japan [31].  Figure 29 shows the measurement setup with CMOS Gaussian monocycle pulse (GMP) transmitter developed by Hiroshima University [32]. Also, Figure 30 shows a GMP equivalent time sampling circuit for confocal imaging developed by a Hiroshima University team [33].

**Figure 31:**
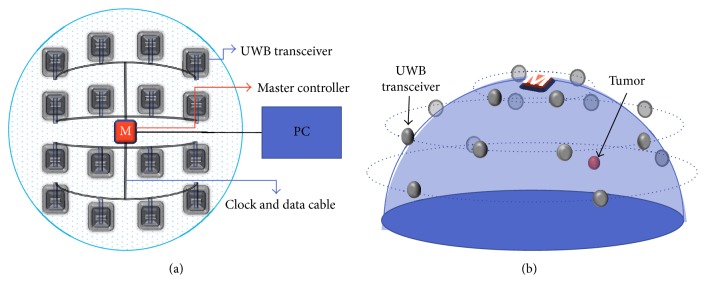
Breast cancer imaging system with 16 UWB transceivers. (a) Configuration of 16 UWB transceivers and master controller and (b) location of 16 UWB transceivers with a 3 mm radius tumor inside [34].

In [34], we proposed an instantaneous microwave imaging system with time-domain measurements. The system consists of a master controller and 16 UWB transceivers.  Figure 31 shows the configuration of the time-domain microwave breast imaging system that we previously presented. It consists of 16 UWB transceivers around a 3D hemisphere, with a master controller at the center. We used CMOS transceiver chips instead of a high-precision pulse generator and high-speed oscilloscope; also, a master controller performs the role of switching matrix. We used the equivalent time sampling technique to achieve a sampling rate of 28.2 Gs/s by 1.76 GHz sampling clock. A single system clock from the master controller drives all the 16 transceivers. The transceivers transmit a UWB pulse one at a time, and the rest of them receive the signal simultaneously, such that the total scan time can be dramatically reduced to 1.32 *µ*sec at 1.76 GHz sampling clock, while a typical scan time with a frequency domain system is more than 10 seconds. As patient movements during measurement cause artifacts in the acquired image, and a fast scan time is very critical for achieving a clear breast image.

**Figure 32:**
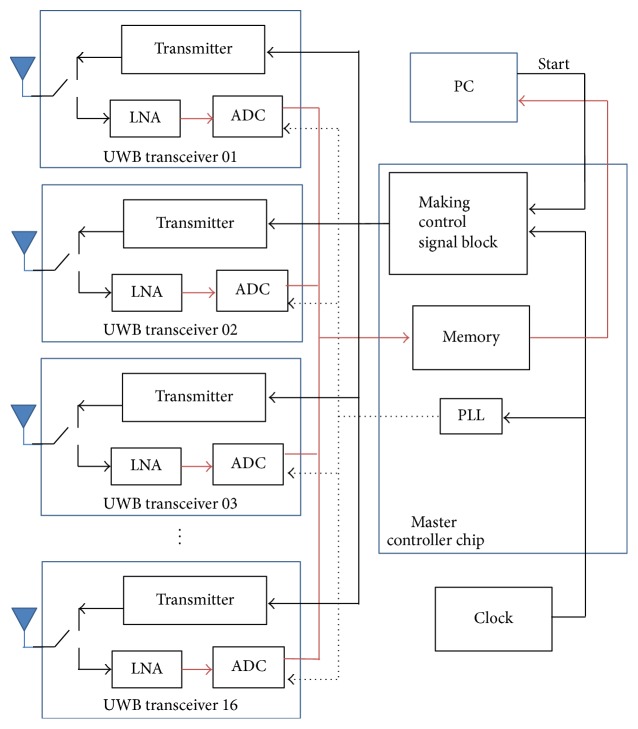
Block diagram of time-domain breast imaging system.

Figure 32 shows our system diagram. The master controller coordinates the operation of 16 UWB transceivers and distributes a global clock to transceivers. Also, it collects all received signals from transceivers and samples them using equivalent time sampling. The collected signals are delivered to a PC for image reconstruction.

The time-domain measurement system suffers from a low signal to noise ratio, because high frequency RF signal attenuates rapidly within breast tissue. Thus, the signals have to be measured repeatedly and averaged to improve SNR, because SNR is proportional to the square root of the number of measurements.

**Figure 33:**
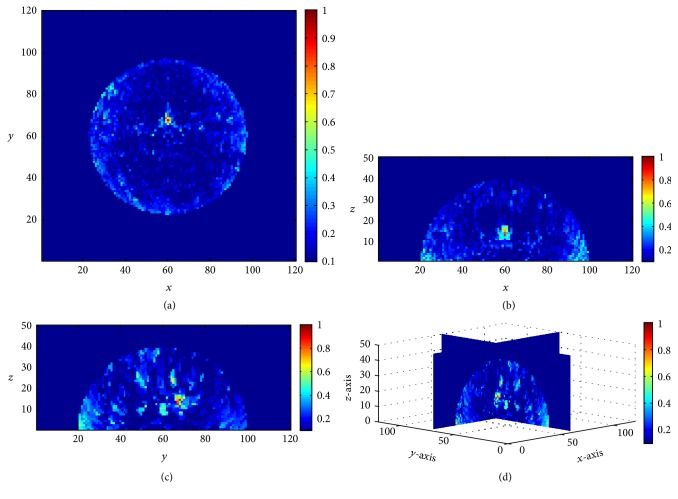
Cross-sectional images of restored breast model with 1,000 measurements, (a) *x*-*y* plane, *z* = 17, (b) *x*-*z* plane, *y* = 70, (c) *y*-*z* plane, *x* = 60, and (d) 3D image [34].

Figure 33 shows the restored image of 3D hemispherical breast model composed of a 2 mm thick skin, fat, and 3 mm radius tumor with 1,000 measurements when all possible noise sources are present, such as clock skew (±1% + 5 ps), clock jitter (1.6 ps), and white noise. The diameter of the breast model is 84 mm and the tumor located at *x* = 70, *y* = 60, and *z* = 17. 16 antennae which are placed along the breast model transmit a modulated Gaussian pulse which is centered 6 GHz with pulse width 0.3 ns.

## 4. Conclusions

Several research groups have studied microwave breast imaging systems that are noninvasive and not harmful to human as alternative early diagnostic breast cancer imaging methods. Besides, microwave imaging system does not require the compression of breast. Unlike initial studies, a large-scale study published in 2007 found that the variation of dielectric properties of the normal breast is large. Further research such as classification is going on to solve heterogeneity in the breast. Several groups have already conducted clinical trials with patients and the results are very promising. In particular, a CMOS chip-based time-domain measurement microwave system can reduce the scan time to within 1 sec when the 1000 repeated signals are averaged with 16 antennae. In the time-domain measurement, it takes 1.32 *µ*sec as once measurement by equivalent time sampling with 1.76 GHz sampling clock. Thus, it can diminish the artifacts caused by patient movements due to long measurement time. In addition, it can be implemented at low prices, because it does not require expensive equipment like VNA, oscilloscope, and pulse generator. The prices of VNA, oscilloscope, and pulse generator are about $10,000~100,000 while a transceiver chip is about $100. Thus, the production cost of our imaging system prototype may be less than $3,000. The development of such a microwave breast imaging system will help young women with dense breast to receive regular breast cancer screening safely.
