# Corrigendum to “Recent Advances in Microwave Imaging for Breast Cancer Detection”

**DOI:** 10.1155/2018/1657073

**Published:** 2018-05-02

**Authors:** Sollip Kwon, Seungjun Lee

**Affiliations:** Department of Electronics Engineering, Ewha Womans University, Seoul, Republic of Korea

In the article titled “Recent Advances in Microwave Imaging for Breast Cancer Detection” [1], Figure 1 has been replaced and Figure 29 has been blanked as the copyright permissions to use them were not obtained. Also, the legends of Figures 1, 2, 3, 4, 5, 6, 7, 8, 9, 10, 11, 12, 13, 14, 15, 17, 18, 19, 20, 21, 22, 23, 24, 25, 26, 27, 28, and 30 have been corrected in place to include the copyright permission.
